# The “architecture of distancing”: a mode of abortion governance illustrated by the Italian case

**DOI:** 10.1080/26410397.2026.2638043

**Published:** 2026-03-02

**Authors:** Debra Lanfranconi

**Affiliations:** PhD candidate, Swiss Graduate School of Public Administration (IDHEAP), University of Lausanne, Lausanne, Switzerland. *Correspondence*: debra.lanfranconi@unil.ch

**Keywords:** abortion rights, abortion access, Italy, conscientious objection, reproductive governance, Law 194/78, architecture of distancing

## Abstract

This commentary introduces the concept of an “architecture of distancing” to describe a mode of reproductive governance that preserves the legal recognition of abortion while constraining the conditions necessary for exercising this right. Using Italy as a paradigmatic case, it illustrates how the law has, from its inception, embedded mechanisms that maintain formal legality yet distance individuals from access – through strict procedural requirements, institutionalised conscientious objection, and the integration of anti-abortion groups into public services. The Italian case demonstrates that obstacles to abortion access are often not mere implementation failures, but features built within even ostensibly liberal legal frameworks, thereby helping illuminate contexts in which abortion is “legal but inaccessible”. More broadly, the concept offers a lens for capturing contemporary forms of reproductive governance that restrict without prohibiting. As there is no architecture without architects, the architecture of distancing draws attention to the political, institutional, and social arrangements that sustain these dynamics and underscores the importance of prioritising the material realisation of reproductive rights over their symbolic recognition.

## Résumé

Ce commentaire introduit le concept d'« architecture de la distanciation » pour décrire un mode de gouvernance reproductive qui maintient la reconnaissance légale de l'avortement tout en restreignant les conditions nécessaires à son accès effectif. En prenant l'Italie comme cas paradigmatique, il montre comment la loi a, dès son adoption, intégré des mécanismes qui préservent la légalité formelle tout en tenant les personnes à distance de l'accès à l'avortement, au moyen d'exigences procédurales strictes, de l'institutionnalisation de l'objection de conscience et de l'intégration de groupes anti-avortement dans les services publics. Le cas italien montre que les obstacles à l'accès à l'avortement ne relèvent pas simplement de défaillances dans la mise en œuvre, mais peuvent être inscrits au cœur même de cadres juridiques apparemment libéraux, ce qui permet d'éclairer des contextes où l'avortement est « légal mais inaccessible ». Plus largement, ce concept offre une grille de lecture des formes contemporaines de gouvernance reproductive qui restreignent sans interdire. Puisqu'il n'y a pas d'architecture sans architectes, l'architecture de la distanciation attire l'attention sur les arrangements politiques, institutionnels et sociaux qui soutiennent ces dynamiques, et souligne l'importance de privilégier la réalisation concrète des droits reproductifs plutôt que leur reconnaissance symbolique.

## Resumen

Este comentario introduce el concepto de “arquitectura de distanciamiento” para describir un modo de gobernanza reproductiva que preserva el reconocimiento legal del aborto a la vez que restringe las condiciones necesarias para ejercer este derecho. Utilizando Italia como caso paradigmático, se ilustra la manera en que la ley, desde su inicio, integró mecanismos que mantienen la legalidad formal, pero distancian a las personas del acceso mediante estrictos requisitos procesales, objeción de conciencia institucionalizada y la integración de grupos antiaborto en servicios públicos. El caso italiano demuestra que los obstáculos al acceso al aborto a menudo no son meras fallas de ejecución, sino características creadas incluso dentro de marcos legislativos ostensiblemente liberales, por lo que contribuye a iluminar los contextos en que el aborto es “legal, pero inaccesible”. De manera más general, el concepto ofrece una perspectiva para capturar formas contemporáneas de gobernanza reproductiva que restringen sin prohibir. Dado que no hay arquitectura sin arquitectos, la arquitectura de distanciamiento dirige la atención hacia las estructuras políticas, institucionales y sociales que sustentan estas dinámicas, y subraya la importancia de priorizar la realización material de los derechos reproductivos por encima de su reconocimiento simbólico.

## Introduction

In many countries, abortion rights do not translate into effective access. Despite legal recognition of this right, individuals seeking to end a pregnancy frequently encounter obstacles. These barriers are often interpreted as implementation failures, assuming that the law is sound but poorly applied and locating explanatory responsibility with frontline workers.

This commentary advances an alternative interpretation. Obstacles to abortion access are not unintended consequences of flawed implementation, but a mode of reproductive governance in which barriers are codified within the legal framework itself. I conceptualise this configuration as an “architecture of distancing”: a specific reproductive governance regime in which access barriers are structural features of the law rather than deviations from it. This lens highlights how ostensibly liberal legal frameworks can recognise a right while simultaneously containing the conditions for its non-realisation.

Such a reframing redirects analytical attention from implementers to architects: the political, institutional, and social actors who tolerate, design and consolidate mechanisms of distancing. It also illuminates contemporary conflicts over the re-regulation of abortion, where legality is not necessarily contested, yet access is restricted by mechanisms of distancing.

To illustrate how an architecture of distancing operates, I focus on Italy, where its dynamics are especially visible and long-standing. Law 194, enacted in 1978,^1^ made voluntary termination of pregnancy legally permissible. Yet, nearly fifty years later, timely and safe access to abortion still often resembles an obstacle course. Prevailing narratives attribute barriers to implementation failures, notably the high prevalence of conscientious objection among healthcare professionals: 60.5% of gynaecologists nationwide, and over 80% in some southern regions, producing what have been described as “abortion deserts”.^2^ By contrast, the analysis of the Italian case reveals that these obstacles arise from – and are consistent with – the very provisions of Law 194/78, which has institutionalised coexistence of formal legality and practical distancing since its adoption.

## Conceptualising the “architecture of distancing” as a mode of reproductive governance

Reproductive governance refers to the mechanisms through which states, religious institutions, NGOs, social movements, and market actors intervene to produce, monitor, and control reproductive behaviours.^3^ Such governance operates “from above”, through policies, laws, and religious doctrines, while also being embedded in clinical practices and popular discourses.^4^ This perspective situates abortion governance within broader political-economic dynamics that serve the agendas of the state, the Church, and other actors.^5^ It broadens the analysis beyond explicit prohibitions, revealing how power relations and actor configurations shape the social, political and institutional processes through which reproductive health policies are translated into practice.^6^ Research using this lens demonstrates that legality can coexist with restrictive forms of reproductive governance. In Turkey, for instance, a governmental pronatalist agenda limits abortion access not via prohibition but through indirect mechanisms such as the withdrawal of public provision and declining family planning services.^7^

Building on this scholarship, I introduce the concept of architecture of distancing to describe a configuration of reproductive governance in which inaccessibility is not peripheral to the law but built into it. This concept captures situations where abortion is legally recognised, yet the material conditions necessary for its exercise are not ensured by the law. The architecture of distancing describes ambivalent – often seemingly liberal – regulatory devices that preserve symbolic legality while organising practical distance from access.

This conceptualisation extends reproductive governance approaches by shifting analytical focus from implementation to the legal architecture that produces barriers. Rather than viewing obstacles as deviations from the law's intentions, it demonstrates how laws can incorporate the very conditions that limit the exercise of reproductive rights.

This shift also relocates responsibility for restricted access. Instead of centring on frontline actors and implementation problems, it directs attention to the political, institutional and social architects who, through law-making, design and consolidate mechanisms that distance formal legality from practical access. It brings into view the legal and institutional arrangements that structure the gap between the symbolic recognition of abortion rights and their material realisation. Finally, this lens sheds light on contemporary conflicts over abortion re-regulation. In many settings, legality remains intact while access is reshaped through incremental legal adjustments that introduce procedural, institutional, or symbolic constraints. These seemingly minor changes distance, rather than prohibit, the exercise of abortion rights. The architecture of distancing captures these dynamics as a contemporary mode of reproductive governance.

## The architecture of distancing at work: the Italian case

The Italian case makes visible how an architecture of distancing can be embedded within legislation that does not secure the material conditions for abortion access.

### The legal foundations of distancing

The origins lie in the text and structure of Law 194/78 itself. Enacted amid intense socio-political polarisation, the law emerged as a negotiated compromise between opposing moral and political forces after more than three years of debates. In 1978, when the law was adopted, Italy was governed by a coalition led by Christian Democracy, a party with strong Catholic roots and the dominant force in Italian politics in the post-war decades. Supported by the Catholic Church, Christian Democracy ideologically opposed abortion's legalisation, while feminist and secular movements, allied with the Radical Party, mobilised for its decriminalisation.^8^

The outcome, in 1978, was a pluralist compromise combining protection of motherhood (enshrined in Article 1) with the conditional possibility of abortion. Abortion was not decriminalised but legalised* within a framework of priority values: the social value of motherhood, responsible procreation, and the protection of human life from its inception.^10^ Praised as a well-balanced law embracing the “different shades of the plurality of values which characterise Italian society” [p. 98],^11^ it is widely regarded as an untouchable compromise, endorsed across political divides. Even right-wing Prime Minister Giorgia Meloni has pledged not to amend it^12^ – a surprising stance given her publicly expressed opposition to abortion.† ^13^ Yet it is precisely this celebrated compromise that has, from the start, incorporated the conditions of distancing between formal legality and effective access.

Symbolically, abortion is tolerated rather than legitimised. The law's title (“Norms on Social Protection of Motherhood and the Voluntary Interruption of Pregnancy”) and its opening articles establish “a rule/exception relationship between the continuation of every pregnancy and abortion” [p. 1037].^12^ This moral framing is coupled with a web of procedural requirements. The law allows only therapeutic, not elective, abortion. Within the first 90 days, the pregnant person must demonstrate that carrying the pregnancy to term would endanger their physical or mental health; obtain medical certification of both the pregnancy and the intention to terminate it; and observe a seven-day reflection period (except in emergencies). Minors require parental consent (or, failing that, judicial authorisation). Abortions may only be performed by gynaecologists in public or accredited facilities, creating a quasi-monopoly over provision in the public health system and a medicalised and hospital-centred model of care.‡ ^12^ From its inception, the law framed abortion not as a subjective right, but as a highly regulated exception within a system privileging motherhood. This compromise institutionalised distancing as a mode of reproductive governance. Morally, it discourages reproductive autonomy; materially, it constructs a sequence of procedural steps that distance those seeking abortion from care.

### Conscientious objection: the primary pillar of distancing

The architecture of distancing embedded in Law 194/78 finds its clearest expression in Article 9, which grants healthcare personnel the right to refuse to perform abortion-related activities on religious or moral grounds. Initially, the conscientious objection clause was seen as aligned with constitutional pluralism^10^ and as a reasonable compromise: “abortion would be broadly legal, but no individual would be compelled to support this service” [p. 283].^17^ Over time, however, what was conceived as an exception to protect a minority has become the prevailing norm.^10,18^

Despite being the only professionals authorised to perform abortions, over 60% of gynaecologists are objectors, with rates exceeding 80% in some regions.^19^ This produces significant regional disparities, overburdens non-objectors, extends waiting times (often beyond four weeks), and forces an estimated 21,000 women annually to travel outside their regions for care.^2,8,20–22^ These burdens fall disproportionately on individuals in lower-income regions or facing other economic disadvantages,^20,23^ amplifying reproductive health inequities.^24^ Indeed, cross-regional reproductive travel reinforces gender, social, and racial inequalities, as mobility depends on citizenship status, information and economic resources.^5^ Service shortages also contribute to recourse to self-managed abortions, often through pills purchased online.^12^ While legal abortion rates have declined since 1978, illegal procedures remain steady, estimated to be between 11,000 and 27,000 per year, or up to 27% of total cases.^25^

Given these tangible impacts, it is unsurprising that debates on abortion access in Italy crystallise around conscientious objection. Yet this focus tends to produce two reductive interpretations. The first frames the problem as one of implementation: Law 194/78 is regarded as sound and balanced but undermined, in practice, by high rates of objectors.§ The second conceptualises access issues as a conflict between two individual rights: reproductive autonomy versus freedom of conscience. Although both perspectives capture substantive elements, they tend to detach conscientious objection from the broader legal and institutional architecture that sustains it. By allowing and protecting objection, Article 9 is a structural pillar of the architecture of distancing that restricts access while leaving formal legality intact.

Importantly, not all objections stem from religious or moral convictions. Non-objectors frequently face heavier workloads, limited career prospects, and exposure to discrimination due to the stigma surrounding abortion.^18,20,28–30^ In such settings, objection functions as a “safety valve”: as the only legal reason to refuse to provide a lawful health service, it can be invoked for heterogeneous motives, not all of which are grounded in moral or religious beliefs.^24^ Italian law facilitates this practice of “convenient objection” [p. 60]^18^: declaring oneself an objector requires neither justification nor the assumption of alternative duties and entails no financial penalties. Objectors are simply exempted from a procedure that otherwise falls within their routine responsibilities.^11^

Consequently, conscientious objection does not operate (solely) as an individual expression of conscience. Its widespread use is also facilitated by a legal design that permits objection without need of justification or any concrete consequences, allowing heterogeneous motivations to converge into a collective and institutionalised pillar of distancing.

### Other components of the architecture of distancing

While conscientious objection is the most visible pillar, the architecture of distancing also relies on provisions that embed anti-abortion actors within *consultori*, public family planning centres conceived as a cornerstone of abortion care. Article 2 of Law 194/78 charges *consultori* to “overcome the factors that might lead the woman to terminate her pregnancy” and allows them to rely on “the voluntary collaboration of suitable grassroots social organisations and volunteer associations” to fulfil this task. These provisions constitute a channel within the law through which anti-abortion actors can intervene in reproductive decision-making. Over time, they enabled regional partnerships with pro-life groups and the establishment of pro-life counselling centres inside public clinics.^31^ In April 2024, the Senate approved an amendment that further strengthened the presence of anti-abortion associations in these centres. Proposed by Brothers of Italy, Prime Minister Meloni's party, the amendment draws directly on Article 2 of Law 194/78. It consolidates the influence of such groups by allowing these partnerships to be funded through state, rather than only regional, resources, and by granting governmental support for their integration into the public healthcare system.^32,33^

This development suggests an evolution in the architecture of distancing: from passive state disengagement in ensuring effective abortion access to active involvement in moral dissuasion. Importantly, this shift does not challenge the law, but operates through – and directly exploits – its provisions. Current dynamics therefore do not represent a rupture, but a continuation of the original logic of a legal framework that recognises abortion as a legal possibility while organising the conditions for its distancing.

Nearly fifty years after the law's adoption, abortion remains “the bone of contention in an ideological battle” [p. 424]^26^ between competing views of life and reproductive autonomy. In an increasingly polarised climate, the distancing between formal legality and actual access risks widening further. The 2024 amendment is indicative of this trend. It has been accompanied by other legislative proposals, including measures to grant embryos legal personhood, recognise the conceived as family members, and establish a national day for the “protection of nascent life”.^34,35^ Although none have been adopted, they signal efforts to strengthen anti-abortion orientations within public policy. As such, they point to a broader dynamic seeking to extend the mechanisms of distancing already present in Italy's abortion governance framework.

## Conclusion

The Italian case reveals a mode of reproductive governance in which abortion remains formally legal yet materially constrained by the very structure of the law – a constellation described as an “architecture of distancing”. The distance between formal legality and material access does not stem from an implementation failure but constitutes a stable feature of the legal framework that, from the outset, institutionalised distancing as a mode of abortion governance. Procedural requirements, conscientious objection and the integration of anti-abortion actors into public services are not misapplications of the law, but components of the architecture of distancing which the law actively enables. Responsibility therefore lies less with individual providers invoking conscientious objection than with the political and institutional architects who shape the conditions under which abortion is legally possible yet practically limited. Distancing is not incidental but the outcome of deliberate governance choices.

From this perspective, the architecture of distancing helps illuminate current conflicts over abortion re-regulation. The *Dobbs* ruling in the United States underscored that progress toward reproductive freedom is not linear.^36^ Over recent decades, transnational anti-abortion coalitions have mounted coordinated resistance to efforts to promote safe and legal abortion.^6^ This “energized movement” [p.24],^36^ promotes strategies that distance abortion access without challenging its legality, through measures such as mandatory waiting periods, biased counselling requirements or the unregulated practice of conscientious objection.^37^ Such strategies seek to institutionalise new architectures of distancing: preserving legality while shifting the boundary of access. Italy offers an early illustration of this mode of governance, in which legality and distancing have long coexisted within the same legal framework.

The concept of architecture of distancing thus provides a transferable lens for examining contemporary struggles over reproductive governance in a global context marked by populist politics and regressive sexual and reproductive health policies.^38^ Recognising these architectures, as well as the actors who construct and extend them, is a first step toward shifting attention from the symbolic recognition of rights to the conditions that warrant their material realisation.

### Author contributions

*CRediT: Debra Lanfranconi: Conceptualization, Investigation, Methodology, Writing – original draft, Writing – review & editing.*

## Footnotes

* As Berer^9^ explains, legalising abortion means retaining it within the law by specifying the circumstances under which it is allowed, whereas decriminalising it entails removing criminal sanctions altogether.

† For example, before being appointed Prime Minister in 2022, she vowed to “give the right to make a different decision to women who think abortion is the only solution”.^13^

‡ Access to medical abortion further illustrates this medicalised model. Although permitted since 2009 and expanded in 2020, when use was extended up to 63 days and provision was authorised outside hospital settings, availability remains uneven. As of 2025, only two of twenty regions allow access without hospitalisation.^12,14^ Most regions still require multiple facility visits and hospital-based monitoring.^15^ This occurs despite evidence showing that first-trimester medical abortion is safe, effective, does not require specialist training and can be provided by non-physician clinicians.^16^

§ This perspective is echoed by scholars, healthcare professionals and advocacy groups. It is articulated, for instance, by providers interviewed in Chavkin et al.,^18^ by Montanari Vergallo,^26^ and by a member of an advocacy organisation cited in Paterlini.^27^

## Provenance statement

This article was not commissioned and went through external peer review.
